# Maduramicin Inhibits Proliferation and Induces Apoptosis in Myoblast Cells

**DOI:** 10.1371/journal.pone.0115652

**Published:** 2014-12-22

**Authors:** Xin Chen, Ying Gu, Karnika Singh, Chaowei Shang, Mansoureh Barzegar, Shanxiang Jiang, Shile Huang

**Affiliations:** 1 Laboratory of Veterinary Pharmacology and Toxicology, College of Veterinary Medicine, Nanjing Agricultural University, Nanjing, Jiangsu Province, P. R. China; 2 Department of Biochemistry and Molecular Biology, Louisiana State University Health Sciences Center, Shreveport, Louisiana, United States of America; 3 Feist-Weiller Cancer Center, Louisiana State University Health Sciences Center, Shreveport, Louisiana, United States of America; Institute of Biochemistry and Biotechnology, Taiwan

## Abstract

Maduramicin, a polyether ionophore antibiotic derived from the bacterium *Actinomadura yumaensis*, is currently used as a feed additive against coccidiosis in poultry worldwide. It has been clinically observed that maduramicin can cause skeletal muscle and heart cell damage, resulting in skeletal muscle degeneration, heart failure, and even death in animals and humans, if improperly used. However, the mechanism of its toxic action in myoblasts is not well understood. Using mouse myoblasts (C2C12) and human rhabdomyosarcoma (RD and Rh30) cells as an experimental model for myoblasts, here we found that maduramicin inhibited cell proliferation and induced cell death in a concentration-dependent manner. Further studies revealed that maduramicin induced accumulation of the cells at G_0_/G_1_ phase of the cell cycle, and induced apoptosis in the cells. Concurrently, maduramicin downregulated protein expression of cyclin D1, cyclin-dependent kinases (CDK4 and CDK6), and CDC25A, and upregulated expression of the CDK inhibitors (p21^Cip1^ and p27^Kip1^), resulting in decreased phosphorylation of Rb. Maduramicin also induced expression of BAK, BAD, DR4, TRADD and TRAIL, leading to activation of caspases 8, 9 and 3 as well as cleavage of poly ADP ribose polymerase (PARP). Taken together, our results suggest that maduramicin executes its toxicity in myoblasts at least by inhibiting cell proliferation and inducing apoptotic cell death.

## Introduction

Maduramicin (also called Yumamycin) is a monovalent glycoside polyether ionophore antibiotic produced through aerobic fermentation by the bacterium *Actinomadura yumaensis*, which was originally isolated from a soil sample from Yuma County, Arizona, USA [1]–[3]. Maduramicin possesses moderate activity against many Gram-positive bacteria, and exhibits a broad spectrum of anticoccidial activity against the most frequently occurring *Eimeria* species in chickens and turkeys [4], [5]. Thus, currently it is primarily used to control coccidiosis in chickens and turkeys (so-called target animals) for fattening [4], [5]. A dose of 5–7 ppm (mg/kg) of maduramicin in feed is recommended in the USA, the European Union, and many other countries, with a withdrawal period of 5 days before slaughter [4], [5]. Higher doses (>10 ppm) of maduramicin in feed can be toxic in both chickens and turkeys [4]–[6]. Besides, since maduramicin is excreted rapidly and mainly as unchanged form in broilers [4], [7], 2.5–6.1 mg/kg of maduramicin in the broiler litter has been noticed [8]. As cattle, sheep and pigs (so-called non-target animals) are more sensitive to maduramicin [4], clinically maduramicin toxicity has been more frequently observed in these animals when fed with the broiler litter as a source of protein and minerals [8]–[13]. Furthermore, some cases of accidental poisoning with maduramicin in humans have been reported [14], [15]. Histopathologically, maduramicin can induce severe myocardial and skeletal muscle lesions [8]–[14]. It has been proposed that the polyether ionophores (including maduramicin, monensin, narasin, salinomycin, semduramicin, and lasalocid) may form lipophilic complexes with cations (particularly Na^+^, K^+^ and Ca^2+^), thereby promoting their transport across the cell membrane and increasing the osmotic pressure in the coccidia, which inhibits certain mitochondrial functions such as substrate oxidation and ATP hydrolysis, eventually leading to cell death in the protozoa [5], [16]. In general, myoblast cells have more mitochondria. It is not clear whether this is related to maduramicin's higher toxicity to skeletal muscle cells. Nevertheless, to our knowledge, the toxic mechanism of maduramicin in myoblast cells of animals and humans remains largely unknown.

Cell division or cell proliferation is essential for growth, development and regeneration of eukaryotic organisms [17]. In animals (including humans), cell proliferation is directly determined by the progression of the cell cycle, which is divided into G_0_/G_1_, S, and G_2_/M phases, and is driven by various cyclin-dependent kinases (CDKs) [17], [18]. A CDK (catalytic subunit) has to bind to a regulatory subunit, cyclin, to become active [18]. Also, Wee1 phosphorylates specific residues (Tyr15 and Thr14) of CDKs, inhibiting CDKs, which is counteracted by CDC25 through dephosphorylation [18]. However, cyclin activating kinase (CAK) phosphorylates CDKs (Thr161), activating CDKs [18]. Furthermore, p21^Cip1^ and p27^Kip1^, two universal CDK inhibitors, can bind a CDK, inhibiting the CDK activity and the cell cycle progression [19]. Cyclin D-CDK4/6 and cyclin E-CDK2 complexes control G_1_ cell cycle progression, whereas cyclin A-CDK2 and cyclin B-CDK1 regulate S and G_2_/M cell cycle progression, respectively [18]. Therefore, disturbing expression of CDKs and/or the regulatory proteins, such as cyclins, CDC25 and CDK inhibitors, may affect cell cycle progression.

Apoptosis is a type of programmed cell death and occurs actively in multicellular organisms under physiological and pathological conditions [20]. Under physiological conditions, it plays an essential role in regulating growth, development and immune response, and maintaining tissue homeostasis [20]. Under pathological conditions (such as viral infection, toxins, etc.), when cells are damaged too severely to repair, they will also undergo apoptosis via caspase-dependent and -independent mechanisms [20]. In response to apoptotic insults, activation of caspases can be initiated through the extrinsic or death receptor pathway and the intrinsic or mitochondrial pathway [21]. The death receptors are members of the tumor necrosis factor (TNF) receptor gene superfamily, which share similar cyteine-rich extracellular domains and have a cytoplasmic “death domain” of about 80 amino acids [22]. Ligands, such as FasL, TNFα, Apo3L, and Apo2L (also named TRAIL), bind to corresponding death receptors, including Fas (also named CD95), TNFR1, DR3, and DR4/DR5, resulting in receptor oligomerization, which in turn leads to the recruitment of specialized adaptor proteins and activation of caspases 8/10, triggering apoptosis [21], [22]. Furthermore, Bcl-2 family members, including anti-apoptotic (e.g. Bcl-2, Bcl-xL, and Mcl-1) and pro-apoptotic proteins (e.g. BAD, BAK, and BAX), are key players in the regulation of mitochondrial-dependent apoptosis [22], [23]. They work together and with other proteins to maintain a dynamic balance between the cell survival and the cell death [23].

Here, for the first time, we show that maduramicin executes its toxicity at least by inhibiting cell proliferation and inducing cell death in myoblasts (C2C12, RD and Rh30). Maduramicin inhibited cell proliferation through accumulating cells at G_0_/G_1_ phase of the cell cycle, and induced caspase-dependent apoptosis in the myoblasts.

## Materials and Methods

### Materials

Maduramicin ammonium (molecular weight = 934.16, purity>97%, by HPLC) were purchased from Santa Cruz Biotechnology (Santa Cruz, CA, USA), dissolved in dimethyl sulfoxide (DMSO) to prepare a stock solution (5 mg/ml), aliquoted and stored at −80°C. Dulbecco's modified Eagle's medium (DMEM) and 0.05% trypsin-EDTA were obtained from Mediatech (Manassas, VA, USA). Fetal bovine serum (FBS) was from Atlanta Biologicals (Lawrenceville, GA, USA). One Solution Cell Proliferation Assay Kit was from Promega (Madison, WI). Cellular DNA Flow Cytometric Analysis Kit was purchased from Roche Diagnostics (Indianapolis, IN, USA). CF488A-Annexin V and Propidium Iodide (PI) Apoptosis Assay Kit was purchased from Biotium (Hayward, CA, USA). Enhanced chemiluminescence solution was from Perkin-Elmer Life Science (Boston, MA, USA). The following antibodies were used: cyclin A, cyclin B1, cyclin D1, cyclin E, CDK1, CDK2, CDK4, CDK6, CDC25A, CDC25B, CDC25C, p21^Cip1^, p27^Kip1^, Rb, p-Rb (S807/811), survivin, Mcl-1, Bcl-2, Bcl-xL, BAX, BAK, BAD, FasL, Fas/CD95, TNFα, TNFR1, TRAIL, DR4, DR5, FLIP S/L, FADD, TRADD, RIP (Santa Cruz Biotechnology, Santa Cruz, CA, USA), cleaved caspase 3, cleaved PARP (Cell Signaling, Beverly, MA, USA), β-tubulin (Sigma, St Louis, MO), goat anti-mouse IgG-horseradish peroxidase, and goat anti-rabbit IgG-horseradish peroxidase (Pierce, Rockford, IL, USA).

### Cell line and culture

Human rhabdomyosarcoma (Rh30 and RD) cell lines (generously provided by Peter J. Houghton, Nationwide Children's Hospital, Columbus, OH) were grown in antibiotic-free RPMI 1640 (Mediatech, Herndon, VA) supplemented with 10% fetal bovine serum (FBS) (Atlanta Biologicals, Lawrenceville, GA). Murine C2C12 myoblasts (#CRL-1771, American Type Culture Collection, Manassas, VA, USA) were cultured in antibiotic-free high glucose (4.5 g/L) DMEM supplemented with 10% FBS and 2 mM glutamine, at 37°C and 5% CO_2_. C2C12 cells were split at 1∶10 and subcultured every 3 days, to ensure that the cells never grew to more than 50% confluence. RD and Rh30 cells were spilt at 1∶12 and subcultured twice per week.

### Cell morphological analysis and cell proliferation assay

Cell morphological analysis and cell proliferation assay were performed as described [24]. Briefly, cells were seeded in 6-well plates at a density of 1×10^4^ cells/well and cultured in the growth medium overnight at 37°C in a humidified incubator with 5% CO_2_. The next day, maduramicin (0–1 µg/ml) was added. After incubation for 5 days, images were taken with an Olympus inverted phase-contrast microscope equipped with the Quick Imaging system. The cells were then trypsinized and enumerated using a Z1 Coulter Counter (Beckman Coulter, Fullerton, CA, USA). Cells treated with the vehicle (DMSO) alone served as a control.

### One solution assay

Cell proliferation was also evaluated using One Solution Cell Proliferation Assay Kit (Promega), as described [24]. Briefly, cells suspended in the growth medium were seeded in a 96-well plate at a density of 1×10^3^ cells/well (in triplicates) and grown overnight at 37°C in a humidified incubator with 5% CO_2_. The next day, maduramicin (0–1 µg/ml) was added. After incubation for 24–72 h, each well was added 20 µl of one solution reagent and incubated for 1 h. Cell proliferation was determined by measuring the OD at 490 nm using a Wallac 1420 Multilabel Counter (PerkinElmer Life Sciences, Wellesley, MA, USA). Cells treated with the vehicle (DMSO) alone were used as a control.

### Trypan blue exclusion assay

Cell viability was evaluated by the trypan blue exclusion assay. Briefly, cells were seeded in 100-mm dishes at a density of 3×10^5^ cells per dish in the growth medium and cultured overnight at 37°C in a humidified incubator with 5% CO_2_. Following treatment with maduramicin for 24–72 h, the cells (including floating and attached cells) were harvested, pelleted, and resuspended in 1 ml of PBS. Then, 1 part of cell suspension was incubated with 1 part of 0.4% trypan blue solution (Sigma) for 3 min at room temperature. Finally, 10 µl of the trypan blue/cell mixture was applied to a hemacytometer, and the unstained (viable) and stained (nonviable) cells were counted separately under a microscope. For each treatment, at least 300 cells (total) were counted, and the percentage of the stained cells will be calculated.

### Cell cycle analysis

Cell cycle analysis was performed, as described previously [25]. Briefly, cells suspended in the growth medium were seeded in 100-mm dishes at a density of 5×10^5^ cells/dish and cultured overnight at 37°C in a humidified incubator with 5% CO_2_. The next day, cells (<20% confluence) were then treated with maduramicin (0–1 µg/ml) for 24 h. Subsequently, the cells were briefly washed with phosphate buffered saline (PBS) and trypsinized. Of note, the control cells were <50% confluent. Cell suspensions were centrifuged at 1,000 rpm for 3 min, and pellets were fixed and stained with the Cellular DNA Flow Cytometric Analysis Kit (Roche Diagnostics). Percentages of cells within each of the cell cycle compartments (G_0_/G_1_, S, or G_2_/M) were determined using a FACSCalibur flow cytometer (Becton Dickinson, San Jose, CA, USA) and ModFit LT analyzing software (Verity Software House, Topsham, ME, USA). Cells treated with the vehicle (DMSO) alone were used as a control.

### Apoptosis assay

Apoptosis assay was performed, as described previously [25]. Briefly, cells were seeded in 100-mm dishes at a density of 2×10^5^ cells/dish in the growth medium and grown overnight at 37°C in a humidified incubator with 5% CO_2_. Cells were treated with maduramicin (0–1 µg/ml) for 72 h, followed by apoptosis assay using CF488A-Annexin V and Propidium Iodide (PI) Apoptosis Assay Kit (Biotium). Flow cytometry was performed using a FACSCalibur flow cytometer (Becton Dickinson). Cells treated with the vehicle (DMSO) alone were used as a control.

### Western blot analysis

Western blotting was performed, as described previously [25]. Briefly, equivalent amounts of proteins (whole cell lysates) were separated on 8–15% sodium dodecyl sulfate-polyacrylamide gels and transferred to polyvinylidene difluoride membranes (Millipore, Bedford, MA, USA). Membranes were blocked with 5% non-fat dry milk (dissolved in PBS containing 0.05% Tween 20) for 1 h at room temperature, and then incubated with primary antibodies overnight at 4°C, followed by probing with appropriate secondary antibodies conjugated to horseradish peroxidase overnight at 4°C. Immunoreactive bands were visualized by using Renaissance chemiluminescence reagent (Perkin-Elmer Life Science). All experiments were repeated at least 3 times. The blots for interested proteins were semi-quantified using NIH Image J software (http://rsb.info.nih.gov/nih-image/) and were normalized using β-tubulin as an internal control.

### Statistical analysis

Results were expressed as mean values ± SE (standard error). Statistical analysis was performed using one-way analysis of variance (ANOVA) followed by post-hoc Dunnett's test for multiple comparisons. A level of *P*<0.05 was considered to be significant.

## Results

### Maduramicin inhibits cell proliferation and reduces cell viability in myoblast cells

**Figure 1:**
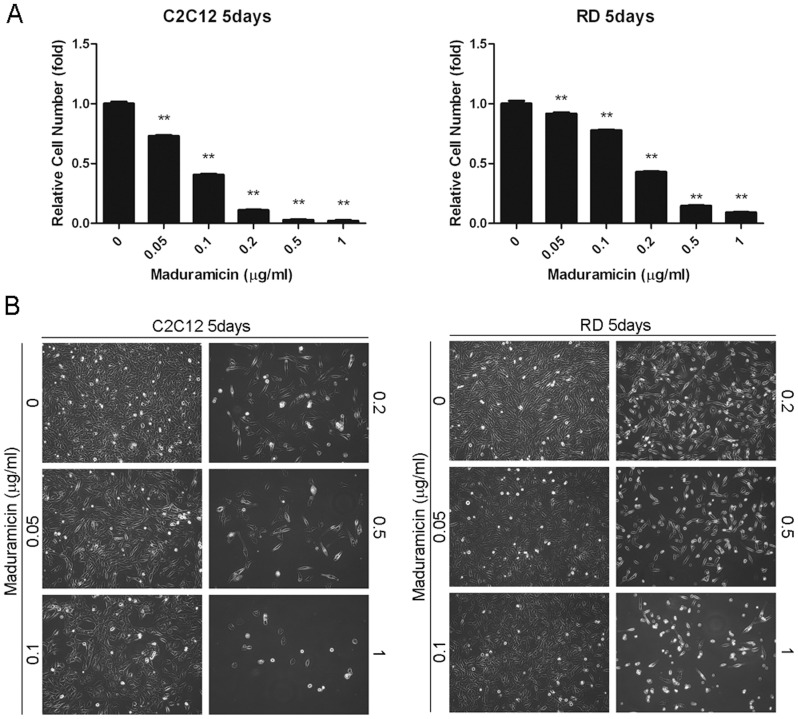
Maduramicin inhibits cell growth in myoblast cells. C2C12 and RD cells (plated in triplicates) were exposed to maduramicin at indicated concentrations for 5 days, followed by cell counting (A) and morphological analysis (B). Data represents mean ± SE (n = 3, corresponding to three independent experiments). **P<0.01, difference with the control group.

**Figure 2:**
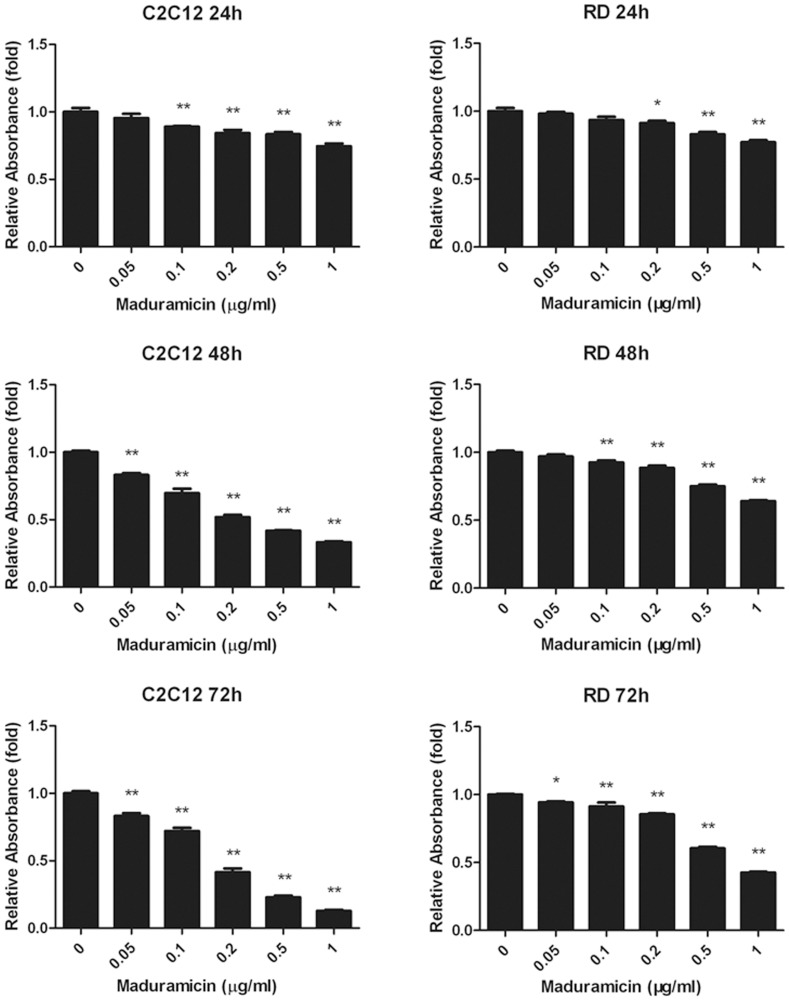
Maduramicin inhibits cell proliferation in myoblast cells. C2C12 and RD cells (plated in triplicates) were exposed to maduramicin at indicated concentrations for 24, 48 or 72 h, followed by one solution assay. Data represents mean ± SE (n = 6, corresponding to six independent experiments). *P<0.05, **P<0.01, difference with the control group.

**Figure 3:**
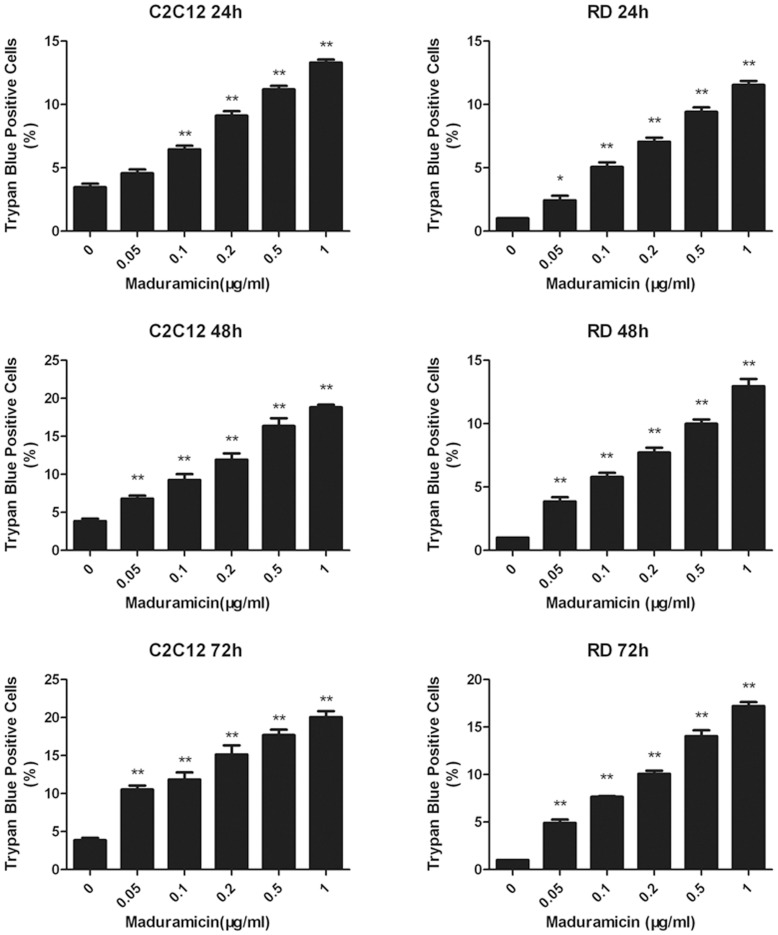
Maduramicin induces cell death in myoblast cells. C2C12 and RD cells (plated in triplicates) were exposed to maduramicin at indicated concentrations for 24, 48 or 72 h, followed by trypan blue exclusion assay. Data represents mean ± SE (n = 3, corresponding to three independent experiments). *P<0.05, **P<0.01, difference with the control group.

To determine the toxicity of maduramicin in skeletal muscle cells, mouse myoblasts (C2C12) and human rhabdomyosarcoma (RD and Rh30) cells were chosen as experimental models. When these cells were treated with maduramicin for 5 days at concentrations of 0–1 µg/ml, followed by cell counting and morphological analysis, we found that maduramicin inhibited cell growth in a concentration-dependent manner (Fig. 1 and S1 Fig.). The IC_50_ values were approximately 0.07, 0.15, and 0.25 µg/ml for C2C12, RD and Rh30 cells, respectively. Similarly, when cells were exposed to maduramicin for 24–72 h, one solution assay also revealed concentration- and time-dependent inhibitory effects on cell proliferation (Fig. 2 and S2A Fig.). In addition, we found that maduramicin also exhibited cytotoxic effects on C2C12, RD (Fig. 3) and Rh30 cells (S2B Fig.), as detected by the trypan blue exclusion assay. When the cells were exposed to maduramicin for 24–72 h, the number of trypan blue positive cells increased in a concentration- and time-dependent manner, suggesting that maduramicin induced cell death. Collectively, our results indicate that maduramicin exerts its toxicity at least by inhibiting cell proliferation and inducing cell death in skeletal muscle cells.

### Maduramicin accumulates myoblast cells at G_0_/G_1_ phase of the cell cycle

**Figure 4:**
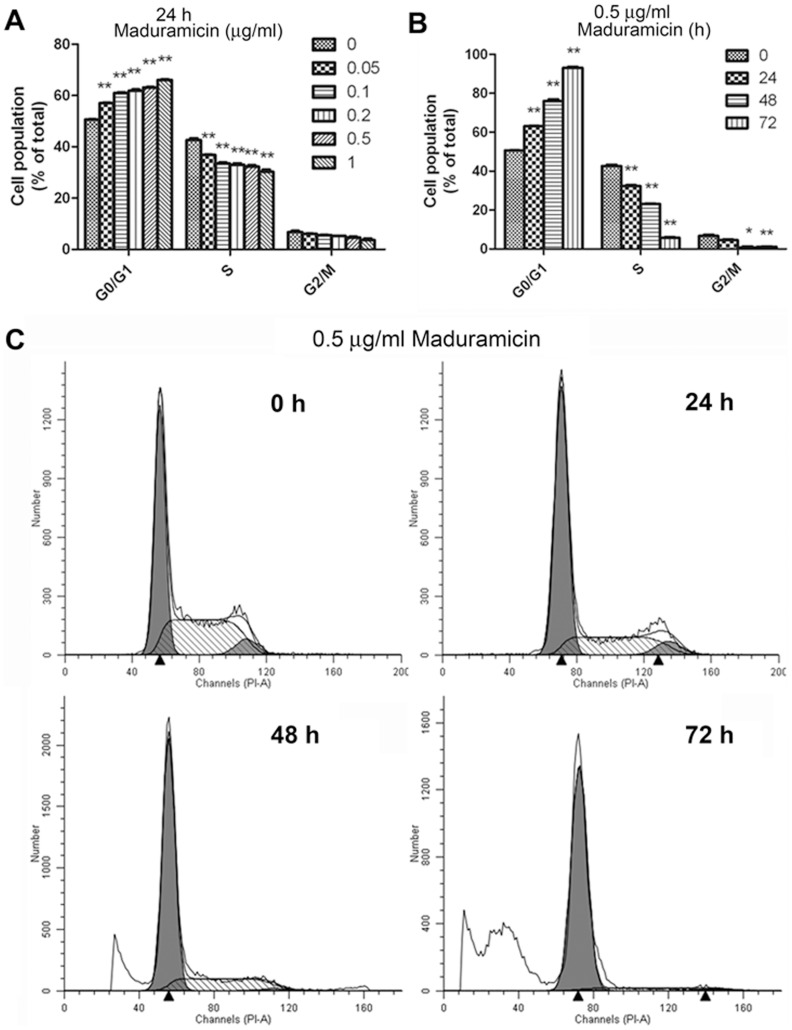
Maduramicin arrests C2C12 cells at G_0_/G_1_ phase of the cell cycle. C2C12 cells were treated with maduramicin for 24 h at indicated concentrations (A), or for indicated time at 0.5 µg/ml (B, C), followed by staining with PI and flow cytometry. (A, B) Results are presented as means ± SE (n = 3, corresponding to three independent experiments). *P<0.05, **P<0.01, difference with the control group. (C) Histograms from a representative experiment show the time-course effect of maduramicin on cell cycle profile in C2C12 cells. Note: Maduramicin increased sub-G_1_ in a time-dependent manner.

To understand how maduramicin inhibits cell proliferation in myoblasts, cell cycle analysis was performed in C2C12 cells. Since the doubling time for C2C12 cells is approximately 19 h [26], C2C12 cells were treated with maduramicin (0–1 µg/ml) for 24 h, followed by PI staining and flow cytometry. As shown in Fig. 4A, treatment with maduramicin accumulated the cells at G_0_/G_1_ phase of the cell cycle in a concentration-dependent manner. Of note, maduramicin at 0.5 µg/ml was able to increase the proportion of the cells in the G_0_/G_1_ phase significantly from approximately 50.6% (control) to 63.0%, and decrease the fraction of the cells in the S phase significantly from about 42.6% (control) to 32.3%, and decrease the portion of the cells in the G_2_/M phase slightly but not significantly from around 6.7% (control) to 4.6%. In addition, maduramicin (0.5 µg/ml) also induced a time-dependent cell cycle arrest at G_0_/G_1_ phase in C2C12 cells (Figs. 4B and 4C). Furthermore, when the cells were exposed to maduramicin for 48–72 h, the percentage of the cells in both of S phase and G_2_/M phase decreased significantly (Figs. 4B and 4C). Noticeably, after treatment with 0.5 µg/ml of maduramicin for 72 h, ∼93% of the cells were in the G_0_/G_1_ phase, 5.8% in the S phase, and 1.1% in the G_2_/M phase, respectively. Moreover, we also observed a time-dependent increase in sub-G_1_ (Fig. 4C), suggesting apoptosis. Together, our results reveal that maduramicin inhibits C2C12 cell proliferation by arresting the cells at the G_0_/G_1_ phase of the cell cycle.

**Figure 5:**
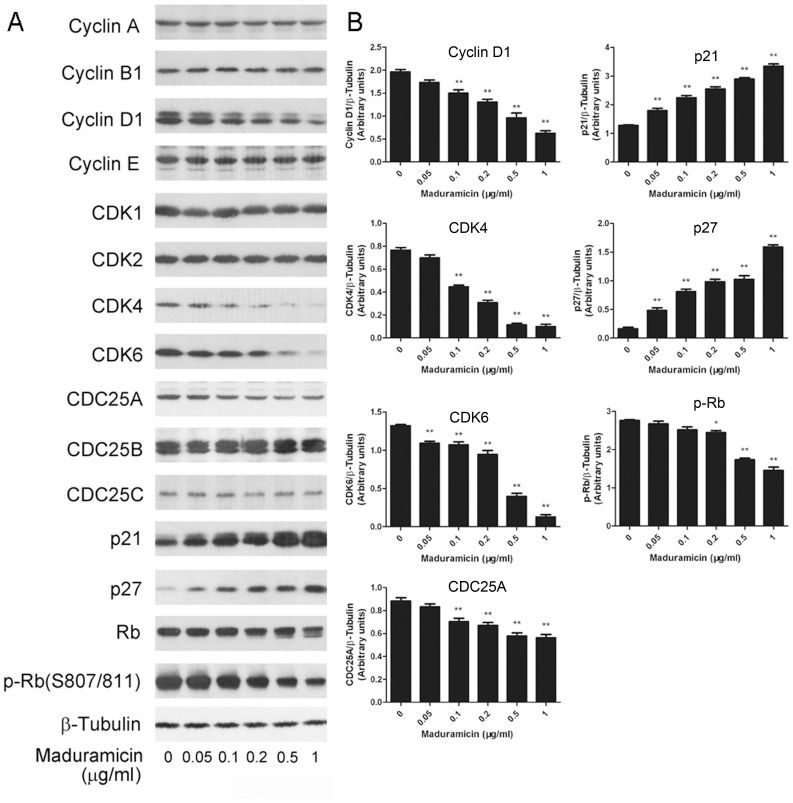
Maduramicin downregulates protein expression of cyclin D1, CDK4, CDK6, and CDC25A, and upregulates expression of p21^Cip1^ and p27^Kip1^, resulting in hypophosphorylation of Rb in C2C12 cells. C2C12 cells were treated with maduramicin for 24 h at indicated concentrations, followed by Western blotting with indicated antibodies. β-Tubulin was used for loading control. Representative blots are shown (A). Blots for indicated proteins were semi-quantified using NIH image J (B). Results are presented as means ± SE (n = 3, corresponding to three independent experiments). *P<0.05, **P<0.01, difference with the control group.

To understand how maduramicin accumulates myoblast cells at G_0_/G_1_ phase of the cell cycle, we examined expression of CDKs and related regulatory proteins, including cyclins, CDC25 and CDK inhibitors in C2C12 cells. As shown in Figs. 5A and 5B, treatment of C2C12 cells with maduramicin for 24 h remarkably inhibited cellular protein expression of cyclin D1, CDK4, and CDK6, and induced expression of p21^Cip1^ and p27^Kip1^ in a concentration-dependent manner. Expression of CDC25A was also significantly downregulated, although the downregulation was modest. Protein levels of other molecules including cyclin A, cyclin B1, cyclin E, CDK1, CDK2, CDC25B and CDC25C were not obviously altered (Fig. 5A). Similar results were observed in RD cells (S3 Fig.). Our results suggest that maduramicin may inhibit expression of cyclin D1, CDK4, CDK6 and CDC25A, resulting in accumulation of myoblasts at G_0_/G_1_ phase of the cell cycle.

As Rb, one of the most important G_1_ phase cyclin/CDK substrates, functions as a regulator of cell cycle progression in the late G_1_ phase [17], we further investigated the effect of maduramicin on Rb phosphorylation. By Western blot analysis, Rb was detected as a 110-kDa band in vehicle-treated control cells. After maduramicin treatment for 24 h, a lower band, which migrates rapidly and represents the dephosphorylated protein, was observed in C2C12 (Fig. 5) and RD cells (S3 Fig.), indicating that maduramicin inhibited phosphorylation of Rb. This was further verified by using the antibodies against specific phospho-Rb (S807/811) (Fig. 5 and S3 Fig.). The data indicate that maduramicin accumulated myoblast cells in G_0_/G_1_ phase of the cell cycle due to inhibition of Rb, a consequence of inhibition of G_1_-cyclin/CDKs.

### Maduramicin induces apoptosis in myoblast cells

**Figure 6:**
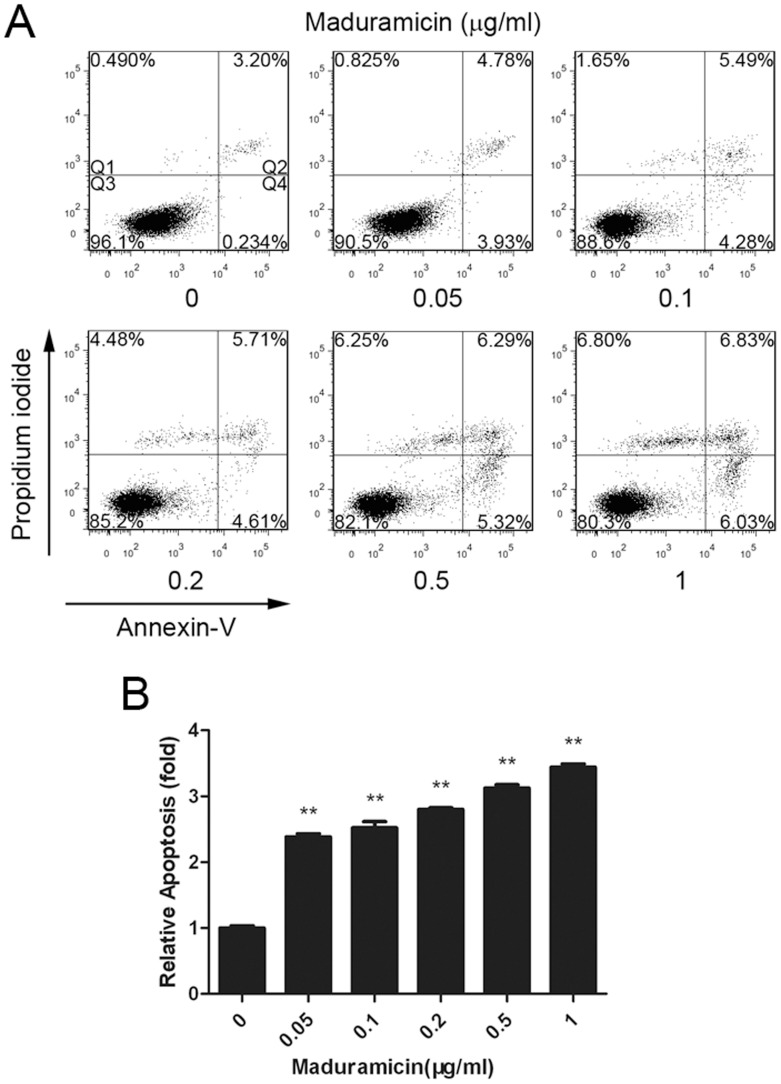
Maduramicin induces apoptosis in C2C12 cells. C2C12 cells were treated with maduramicin for 72 h at indicated concentrations, followed by Annexin V-PI staining and flow cytometry. (A) Histograms from a representative experiment show the apoptotic effect of maduramicin on C2C12 cells. The percentages of necrotic, late apoptotic, viable, and early apoptotic cells are displayed in Q1, Q2, Q3 and Q4, respectively. (B) Bar graphs show that maduramicin induced apoptosis of C2C12 cells in a concentration-dependent manner. Quantitative results (Q2+Q4) are displayed as fold change compared with control. Data represents mean ± SE (n = 3, corresponding to three independent experiments). **P<0.01, difference with the control group.

As maduramicin was able to induce cell death (Fig. 3 and S2B Fig.), and particularly treatment with maduramicin for 72 h resulted in a remarkable increase in sub-G_1_ (Fig. 4C), to further determine if the cell death is due to apoptosis, we carried out Annexin V-PI staining, a conventional approach to detect apoptosis [22], [27]. As shown in Fig. 6, treatment with maduramicin for 72 h induced apoptosis of C2C12 cells in a concentration-dependent manner. Maduramicin at 0.05–1 µg/ml increased the proportion (Q2+Q4) of cells positive for Annexin-V/PI by approximately 2.5–3.5 fold, compared to the vehicle control.

**Figure 7:**
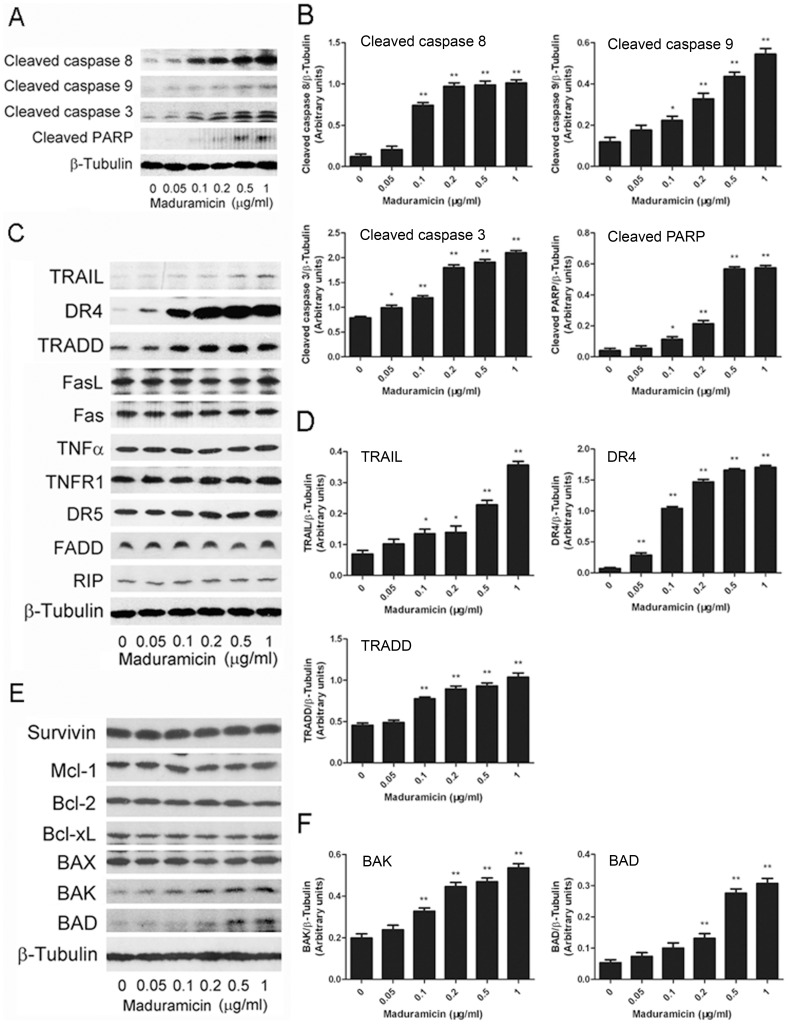
Maduramicin upregulates expression of DR4, TRADD, TRAIL, BAK and BAD, leading to activation of caspases 8, 9 and 3 as well as cleavage of PARP in C2C12 cells. C2C12 cells were treated with maduramicin for 24 h at indicated concentrations, followed by Western blotting with indicated antibodies. β-Tubulin was used for loading control. Representative blots are shown (A, C and E). Blots for indicated proteins were semi-quantified using NIH image J (B, D and F). Results are presented as means ± SE (n = 3, corresponding to three independent experiments). *P<0.05, **P<0.01, difference with the control group.

In addition, we observed that maduramicin induced a concentration-dependent cleavage of PARP, a hallmark of caspase-dependent apoptosis [20], in C2C12 (Fig. 7A) and RD cells (S4A Fig.). As the cleavage of PARP is a consequence of activation of caspases [20], we further investigated whether maduramicin activates the caspase cascade. As expected, maduramicin was indeed able to activate the initiator caspases (caspase 8 and caspase 9) as well as the effector caspase (caspase 3), as judged by increased cleavages of caspases 8, 9 and 3 by Western blot analysis (Fig. 7A and S4A Fig.).

Since the activation of caspase 8 is attributed to activation of the extrinsic pathway [21], [22], we further examined whether maduramicin affects expression of the best-characterized ligands/death receptors (FasL/Fas, TNFα/TNFR1, TRAIL/DR4 and TRAIL/DR5) as well as adaptor proteins (FADD, TRADD, and RIP). Interestingly, treatment with maduramicin for 24 h did not affect the expression of FasL, Fas/CD95, TNFα, TNFR1, DR5, FADD and RIP, but induced a concentration-dependent expression of TRAIL, DR4, and TRADD significantly in both C2C12 (Fig. 7B) and RD cells (S4B Fig.).

In addition, we found that treatment with maduramicin for 24 h did not affect expression of anti-apoptotic proteins, such as survivin, Mcl-1, Bcl-2 and Bcl-xL, but markedly induced expression of pro-apoptotic proteins, including BAK and BAD, in a concentration-dependent manner in C2C12 (Fig. 7C) and RD cells (S4B Fig.). Collectively, the results suggest that maduramicin may induce caspase-dependent apoptosis in myoblast cells through both extrinsic and intrinsic pathways.

### Maduramicin-induced cell death through caspase-dependent and -independent manner in myoblast cells

**Figure 8:**
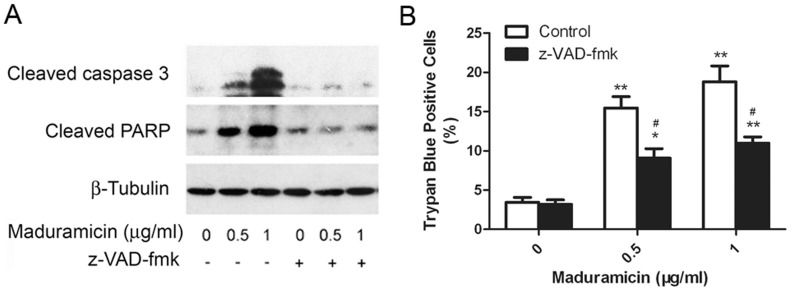
Maduramicin induces caspase-dependent apoptosis in C2C12 cells. C2C12 cells (plated in triplicates), pretreated with or without z-VAD-fmk (20 µM) for 1 h, were treated with maduramicin for 24 h at indicated concentrations, followed by Western blotting with indicated antibodies (A), or for 48 h at indicated concentrations, followed by trypan blue exclusion assay (B). Data represents mean ± SE (n = 3, corresponding to three independent experiments). ^*^ *P*<0.05, ^**^ *P*<0.01, difference with the control group. ^#^ *P*<0.05, difference with z-VAD-fmk group.

To determine whether maduramicin induces cell death is completely through caspase-dependent mechanism, benzyloxycarbonyl-Val-Ala-Asp (OMe) fluoromethylketone (z-VAD-fmk), a pan-caspase inhibitor, was utilized. Pretreatment with z-VAD-fmk (20 µM) for 1 h almost completely blocked maduramicin-induced activation of the caspases, as maduramicin-induced cleavage of PARP, a substrate of caspases [20], was almost completely prevented in C2C12 cells (Fig. 8A). However, pretreatment with z-VAD-fmk only partially prevented maduramicin-induced cell death, as detected by the trypan blue exclusion assay (Fig. 8B). Therefore, the results suggest that maduramicin may induce cell death of myoblast cells via caspase-dependent and -independent mechanisms.

## Discussion

Maduramicin, as an anticoccidial agent, is considerably more potent than other polyether ionophores [5]. However, it is also more toxic than other ionophores [7]. Clinical symptoms, such as watery diarrhea, depression, dullness and ruffled feathers, were observed from the second week in chickens given the feed containing 10 mg/kg of maduramicin or from the third week in birds given the feed containing 5 mg/kg of maduramicin [6]. Also, depression of body weight gain was seen in turkeys when given the feed containing>10 mg/kg of maduramicin [4]. Importantly, as maduramicin is pharmacokinetically excreted mainly as unchanged form in broilers [4], [7], more toxic cases actually occur in non-targeted animals (e.g. cattle, sheep, and pig), when the poultry litter was fed as a source of protein and minerals [6]–[11]. Deleterious effects of maduramicin have been described in cattle fed with poultry litter containing maduramicin even at a final concentration as low as 2.5 mg/kg litter [8]. Also, 37.5 mg/kg of maduramicin in feed is lethal to pigs [9]. Accidental intoxication by maduramicin has also been reported in humans [14]. Pathologically, maduramicin can induce severe skeletal muscle lesion associated with rhabomyolysis, and focal degenerative cardiomyopathy leading to congestive heart failure [8]–[14]. However, to date, the molecular mechanism by which maduramicin induces degeneration of myoblasts is not clear. In this study, for the first time, we present evidence that maduramicin exerts its toxicity to myoblast cells (C2C12, RD and Rh30) by inhibiting cell proliferation and inducing cell death.

The concentrations of maduramicin in skeletal muscle can reach 0.084±0.016 mg/kg (i.e. about 0.1 µg/g) in chickens immediately after fed 7 mg/kg of maduramicin in feed [4]. If the chickens were fed the feed containing 10 mg/kg of maduramicin, the concentration of maduramicin in skeletal muscle would be>0.1 µg/g. Also, non-target animals, such as cattle, sheep, and pigs, are more sensitive to maduramicin [4], [8], [9]. To better mimic the *in vivo* scenario in skeletal muscle, in this study, 0.05–1 µg/ml of maduramicin was used for the *in vitro* studies.

Here we found that maduramicin inhibited cell proliferation (Fig. 1 and Fig. 2) by arresting the cells in G_0_/G_1_ phase of the cell cycle in myoblast cells (Fig. 4). This was associated with downregulating expression of proteins related to the G_1_/S cell cycle progression such as cyclin D1, CDK4, CDK6, and CDC25A, and upregulating expression of CDK inhibitors (p21^Cip1^ and p27^Kip1^), leading to dephosphorylation of Rb (Fig. 5 and S3 Fig.). Similar anti-proliferative effects have also been documented for other polyether ionophores such as monensin and salinomycin [28]–[33]. For instance, monensin inhibits proliferation by inducing G_1_ and/or G_2_/M arrest in human cancer cells, such as CA46 lymphoma cells, SNU-C1 colon cancer cells, ACHN renal cell carcinoma cells, and NCI-H929 myeloma cells [28]–[31]. Salinomycin induces G_2_ arrest in cholangiocarcinoma (Mz-ChA-1 and TFK-1) and hepatocellular carcinoma (SMMC-7721 and BEL-7402) cells, and G_1_ arrest in cholangiocarcinoma EGI-1 and hepatocellular carcinoma HepG2 cells, respectively [32], [33]. Also, salinomycin sensitizes radiation-induced apoptosis by inducing G_2_ arrest in breast cancer cells (MCF-7 and Hs578T) [34]. Moreover, monensin and salinomycin causes accumulation of the coccidian *Toxoplasma gondii* at a late S phase of the cell cycle [35]. Mechanistically, monensin decreases the levels of CDK4 and cyclin A proteins in CA46 lymphoma cells [28]; decreases the levels of CDK2, CDK4, CDK6, cyclin D1 and cyclin A proteins, and increases the level of p27^Kip1^ in SNU-C1 colon cancer cells [29]; decreases the levels of CDK2, CDK6, cdc2, cyclin A and cyclin B1 proteins, and increases the levels of p21^Cip1^ and p27^Kip1^ proteins in ACHN renal cell carcinoma cells [30]; and decreases the levels of CDK2, CDK6, cdc2, cyclin A, cyclin B1, cyclin D1 and cyclin E proteins, and increases increased the level of p21^Cip1^ protein in NCI-H929 myeloma cells [31]. Salinomycin reduces cyclin D1 level in Hs578T breast cancer cells [36]. Apparently, the effects of those ionophores on cell cycle profile are, to some extent, different from each other, depending on different cell lines and experimental conditions used. Although our recent data revealed that maduramicin also induced G_1_ arrest in MDA-MB-231 breast cancer cells (data not shown), further studies are required to determine whether maduramicin can induce cell cycle arrest in S phase and/or G_2_/M phase in other cell lines or under other experimental conditions.

Cyclin D1 can be regulated at transcriptional, translational and post-translational levels, by the downstream pathway of mitogen receptors through the Ras-extracellular signal-regulated kinases (ERKs) and phosphatidylinositide 3-kinase (PI3K) pathways [37]–[39]. In response to mitogen stimulations, both Ras-ERKs and PI3K-Akt pathways can be activated. The activated ERKs can activate the downstream transcription factors c-Myc and AP-1, which, in turn, stimulate the transcription of the CDK4, CDK6 and cyclin D genes [37]. Further, Akt-mediated activation of NF-κB can also upregulate transcription of cyclin D1 [37]. In addition, the mammalian target of rapamycin (mTOR), downstream of PI3K, positively regulates cyclin D1 protein synthesis [38]. Activated Akt can also phosphorylate and inactivate glycogen synthase kinase 3β (GSK3β), which prevents GSK3β from phosphorylating cyclin D (Threonine 286), thereby reducing cyclin D1 degradation [39]. In this study, we found that maduramicin downregulated protein expression of cyclin D1, CDK4 and CDK6. Whether this is attributed to inhibition of ERKs and Akt remains to be determined.

In the present study, we also found that maduramicin was able to induce apoptotic cell death in myoblast cells (Fig. 6), through extrinsic and intrinsic pathways (Fig. 7 and S4 Fig.). This is supported by the findings that treatment with maduramicin for 24 h induced a concentration-dependent increase in protein expression of the death receptor DR4, the ligand TRAIL, as well as TRADD, an adaptor protein for the death receptor TNFR1 [21], [22]. Besides, maduramicin also induced expression of pro-apoptotic BAK and BAD proteins related to intrinsic apoptosis. Activation of both extrinsic and intrinsic pathways resulted in activation of caspases 8, 9 and 3, increasing the cleavage of PARP. Maduramicin did not obviously alter the expression of other proteins related to extrinsic apoptosis, such as FasL, Fas/CD95, TNFα, TNFR1, DR5, FADD and RIP, and the expression of anti-apoptotic proteins associated with intrinsic apoptosis, including survivin, Mcl-1, Bcl-2 and Bcl-xL, in C2C12 (Fig. 7) and RD cells (S4 Fig.). The apoptotic mechanism of maduramicin is somehow in contrast to that of salinomycin or menensin. It has been demonstrated that salinomycin induces apoptosis in a spectrum of human cancer cells, including human CD4+ T-cell leukemia cells, Molt-4 cells, Jurkat cells, Namalwa Burkitt lymphoma cells, MES-SA/Dx5 uterine sarcoma cells, cholangiocarcinoma cells (Mz-ChA-1 and TFK-1), which is independent of caspase activation [32], [40]. However, salinomycin can trigger caspase-dependent apoptosis involving caspases 12, 9 and 3 in dorsal root ganglia as well as Schwann cells [41]. Also, salinomycin induces apoptosis in cisplatin-resistant human colorectal cancer cells (Cisp-resistant SW620 cells) by increasing the protein expression of caspases 3, caspase 8, caspase 9 and BAX, but decreasing the protein expression of Bcl-2 [41]. Furthermore, salinomycin induces apoptosis in hepatocellular carcinoma cells (HepG2, SMMC-7721, and BEL-7402) by decreasing the level of the anti-apoptotic protein Bcl-2 and increasing the level of the pro-apoptotic protein BAX [33]. In addition, monensin induces apoptosis in Caki-2 renal cell carcinoma cells by decreasing the protein expression of Bcl-2 and Bcl-xL, and activating caspases 9, 3, and 7 [30]. In NCI-H9292 myeloma cells, monensin also reduces expression of Bcl-2 protein, and activates caspase 3 [31]. Therefore, the apoptotic mechanism of maduramicin appears to differ from that of salinomycin or monensin. Further investigation may unveil whether this is due to different cell lines used.

Since salinomycin can induce apoptosis in human cancer cells through a caspase-independent mechanism [32], [42], we were interested in investigating whether maduramicin-induced apoptosis is fully caspase-dependent. For this, a pan-caspase inhibitor z-VAD-fmk was utilized. Interestingly, z-VAD-fmk (20 µM) almost completely blocked maduramicin-induced expression of cleaved caspase 3 and cleaved PARP, but only partially prevented maduramicin induced cell death in C2C12 cells (Fig. 8). Our data imply that maduramicin may induce apoptosis of C2C12 cells through caspase-dependent and -independent mechanisms. Apoptosis inducing factor (AIF) is a caspase-independent death effector, which, upon an apoptotic insult, translocates from its normal localization, the mitochondrial intermembrane space, to the nucleus [43]. AIF may induce caspase-independent apoptosis by causing DNA fragmentation and chromatin condensation, and regulating the permeability of the mitochondrial membrane [43]. It has been demonstrated that AIF could maintain the apoptogenic ability in the presence of the pan-caspase inhibitor z-VAD-fmk [44]. As maduramicin was able to induce significant cell death in C2C12 cells, even in the presence of z-VAD-fmk (Fig. 8), possibly maduramicin may induce caspase-independent apoptosis by triggering AIF nuclear translocation in C2C12 cells. Definitely, further research is needed to confirm this. In addition, we noticed that maduarmicin (0.2–1 µg/ml) increased necrosis by approximately 9–14 fold (see Q1, control versus maduramicin treatments, Fig. 6A). Necroptosis has been recently identified as a programmed form of necrotic cell death, which is also caspase-independent [45]. More studies may reveal whether and how maduramicin induces necroptosis. Furthermore, it would also be interesting to determine whether maduramicin can induce autophagy, which may contribute to caspase-independent cell death as well.

In conclusion, this study has demonstrated that maduramicin inhibited cell proliferation and induced apoptosis in myoblast cells (C2C12, RD and Rh30). Mechanistically, maduramicin inhibited cell proliferation by arresting cells in G_0_/G_1_ phase of the cell cycle. This was related to downregulation of cyclin D1, CDK4, CDK6 and CDC25A, and upregulation of CDK inhibitors p21^Cip1^ and p27^Kip1^, resulting in hypophosphorylation of Rb. Maduramicin induced apoptosis through both extrinsic and intrinsic pathways by upregulating expression of TRAIL, DR4, TRADD, BAK, and BAD, leading to activation of caspases 8, 9 and 3 as well as cleavage of PARP. Maduramicin also induced caspase-independent apoptosis, but more studies are required to elucidate the underlying mechanism.

## Supporting Information

S1 Fig **Maduramicin inhibits cell growth in Rh30 cells.** Rh30 cells (plated in triplicates) were exposed to maduramicin at indicated concentrations for 5 days, followed by cell counting (A) and morphological analysis (B). Data represents mean ± SE (n = 3, corresponding to three independent experiments). *P<0.05, **P<0.01, difference with the control group.
(TIF)

S2 Fig **Maduramicin inhibits cell proliferation and induces cell death in Rh30 cells.** Rh30 cells (plated in triplicates) were exposed to maduramicin at indicated concentrations for 24, 48 or 72 h, followed by one solution assay (A) and trypan blue exclusion assay (B). Data represents mean ± SE (n = 6 for one solution assay, n = 3 for trypan blue exclusion assay, corresponding to six and three independent experiments, respectively). *P<0.05, **P<0.01, difference with the control group.
(TIF)

S3 Fig **Maduramicin downregulates protein expression of cyclin D1, CDK4, CDK6, and CDC25A, and upregulates expression of p21^Cip1^ and p27^Kip1^, leading to hypophosphorylation of Rb in RD cells.** RD cells were treated with maduramicin for 24 h at indicated concentrations, followed by Western blotting with indicated antibodies. β-Tubulin was used for loading control.
(TIF)

S4 Fig **Maduramicin upregulates expression of TRAIL, DR4, TRADD, BAK and BAD, leading to activation of caspases 8, 9 and 3 as well as cleavage of PARP in RD cells.** RD cells were treated with maduramicin for 24 h at indicated concentrations, followed by Western blotting with indicated antibodies. β-Tubulin was used for loading control.
(TIF)
