# Supplementary figures and images for: Maduramicin Inhibits Proliferation and Induces Apoptosis in Myoblast Cells
Source: PLoS One. 2014 Dec 22;9(12):e115652. doi: 10.1371/journal.pone.0115652 (PMC4274093; doi:10.1371/journal.pone.0115652)

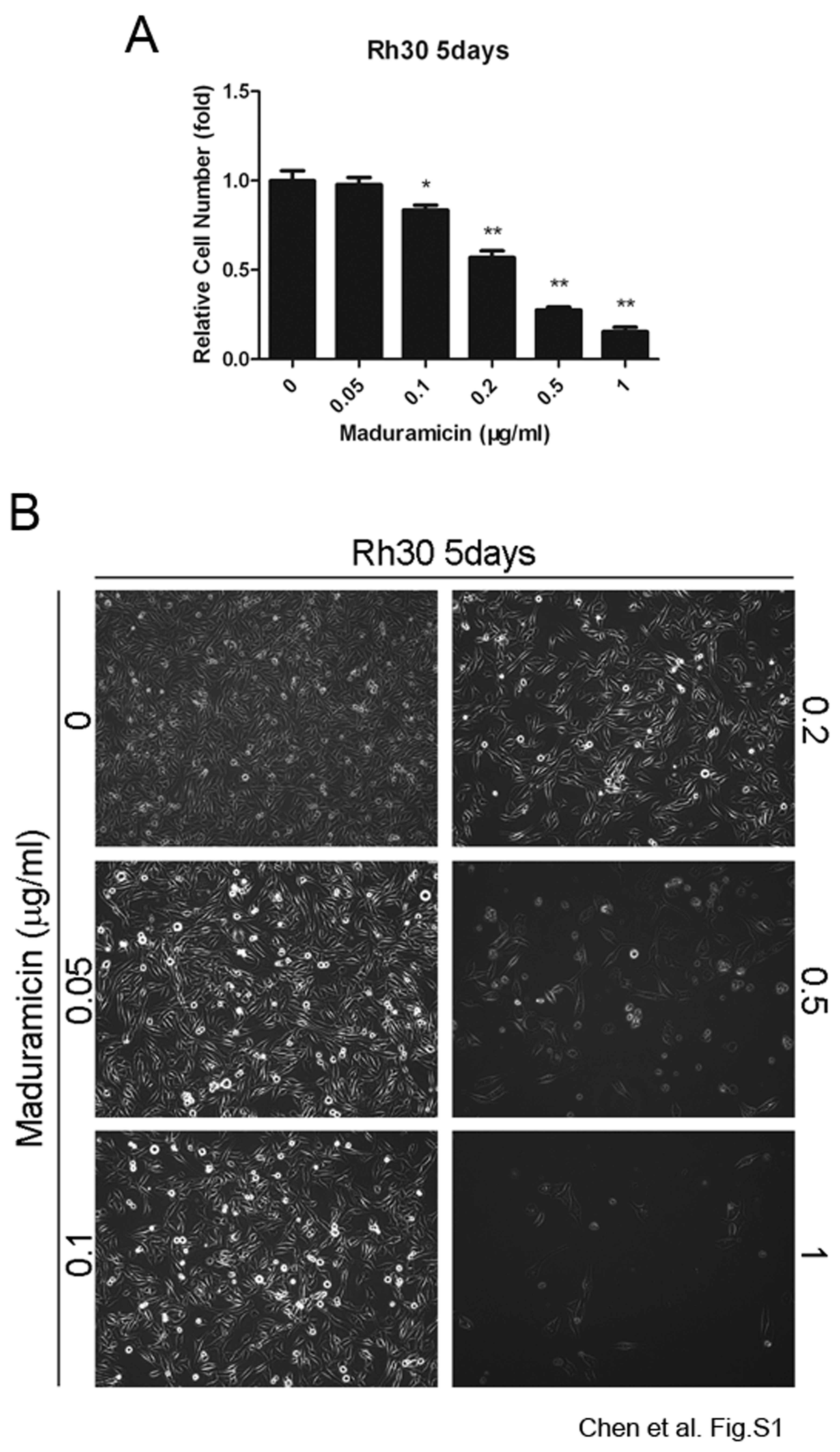

Supplement: S1 Fig — Maduramicin inhibits cell growth in Rh30 cells. Rh30 cells (plated in triplicates) were exposed to maduramicin at indicated concentrations for 5 days, followed by cell counting (A) and morphological analysis (B). Data represents mean ± SE (n = 3, corresponding to three independent experiments). *P<0.05, **P<0.01, difference with the control group. (TIF) [file pone.0115652.s001.tif]

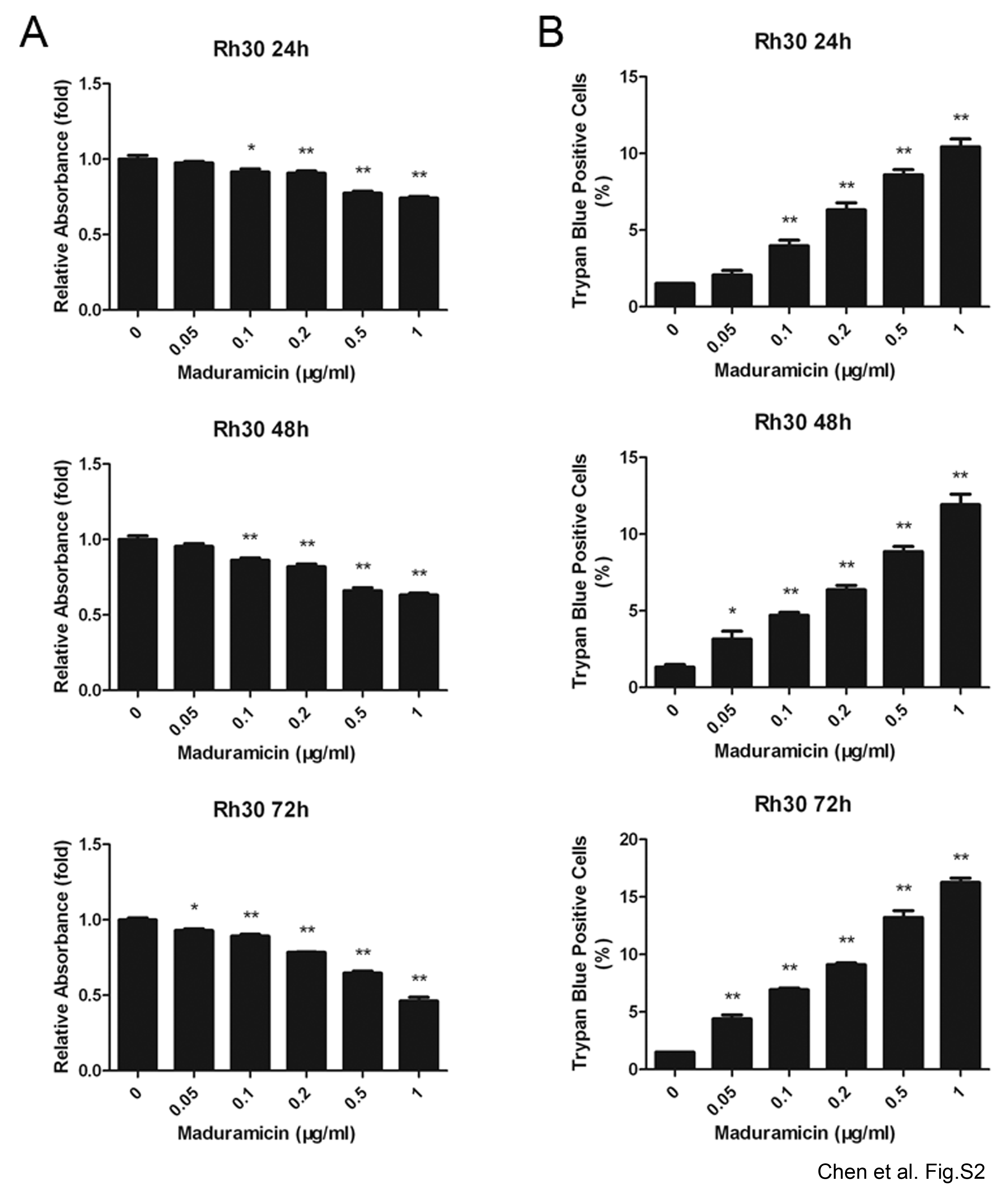

Supplement: S2 Fig — Maduramicin inhibits cell proliferation and induces cell death in Rh30 cells. Rh30 cells (plated in triplicates) were exposed to maduramicin at indicated concentrations for 24, 48 or 72 h, followed by one solution assay (A) and trypan blue exclusion assay (B). Data represents mean ± SE (n = 6 for one solution assay, n = 3 for trypan blue exclusion assay, corresponding to six and three independent experiments, respectively). *P<0.05, **P<0.01, difference with the control group. (TIF) [file pone.0115652.s002.tif]

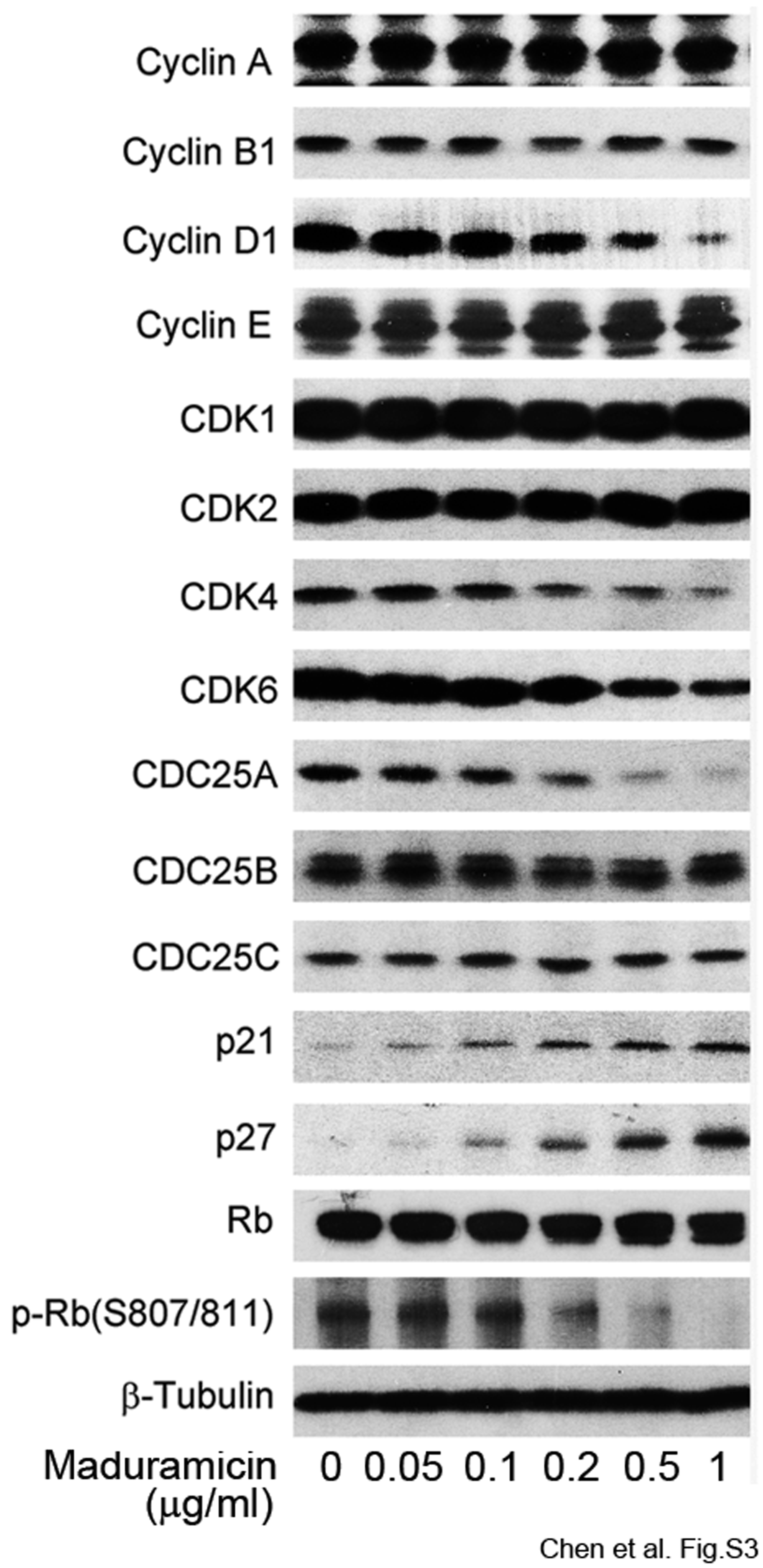

Supplement: S3 Fig — Maduramicin downregulates protein expression of cyclin D1, CDK4, CDK6, and CDC25A, and upregulates expression of p21Cip1 and p27Kip1, leading to hypophosphorylation of Rb in RD cells. RD cells were treated with maduramicin for 24 h at indicated concentrations, followed by Western blotting with indicated antibodies. β-Tubulin was used for loading control. (TIF) [file pone.0115652.s003.tif]

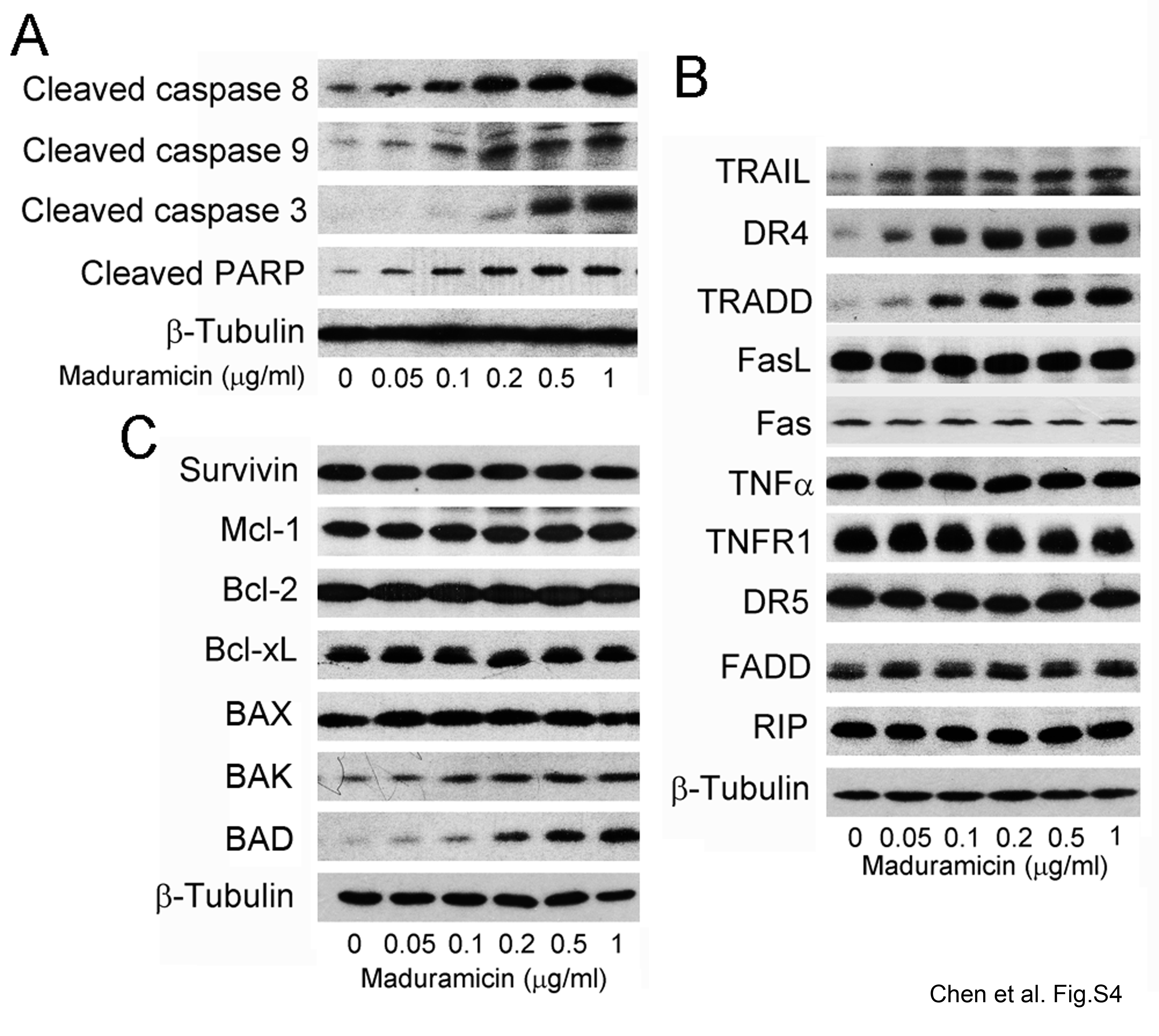

Supplement: S4 Fig — Maduramicin upregulates expression of TRAIL, DR4, TRADD, BAK and BAD, leading to activation of caspases 8, 9 and 3 as well as cleavage of PARP in RD cells. RD cells were treated with maduramicin for 24 h at indicated concentrations, followed by Western blotting with indicated antibodies. β-Tubulin was used for loading control. (TIF) [file pone.0115652.s004.tif]
